# Supplementary material for: Extracellular adenosine deamination primes tip organizer development in Dictyostelium
Source: eLife. 2025 Dec 17;14:RP104855. doi: 10.7554/eLife.104855 (PMC12711200; doi:10.7554/eLife.104855)
Supplement: Figure 1—source data 1. [file elife-104855-fig1-data1.zip › Figure_1_Source_data_1.pdf]

Figue 1-Source data 2_Gel image with labelled bands

Mutant validation

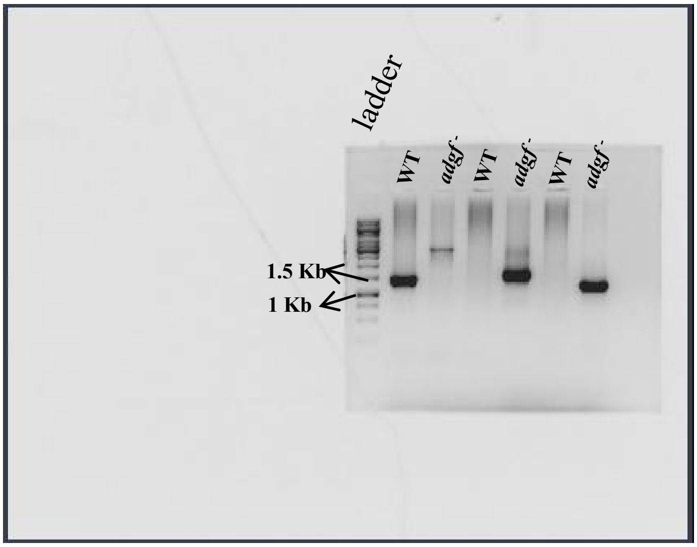

rnlA expression

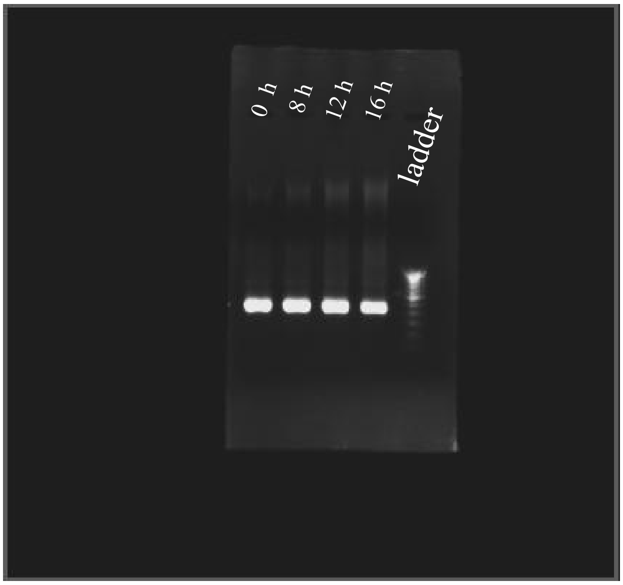

adgf expression

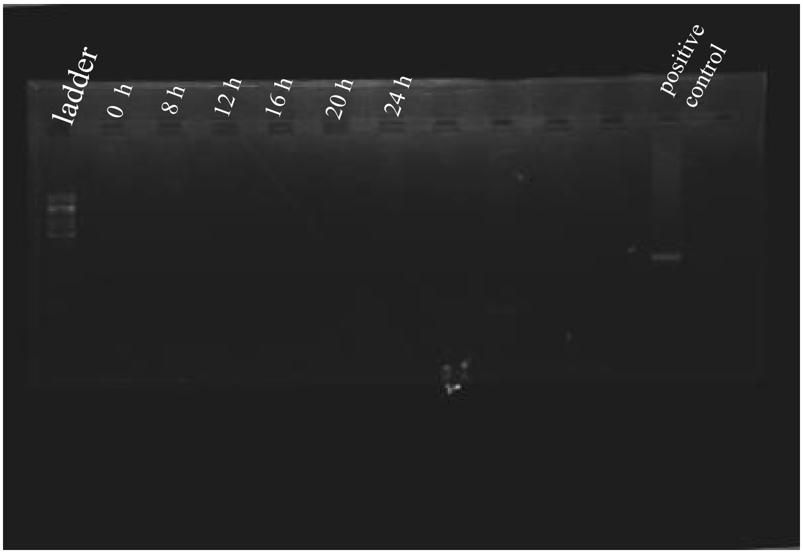

Original gels corresponding to Figure 1, panels B and C. Thermo Fisher ladders were used. The lower gel shows *adgf* expression in the *adgf*⁻ mutant at 0 h, 8 h, 12 h, and 16 h (lanes 1–4), followed by samples from 20 h and 24 h (lanes 5–6), and a positive control (not included in the figure).
