# Supplementary figures and images for: Extracellular adenosine deamination primes tip organizer development in Dictyostelium
Source: eLife. 2025 Dec 17;14:RP104855. doi: 10.7554/eLife.104855 (PMC12711200; doi:10.7554/eLife.104855)

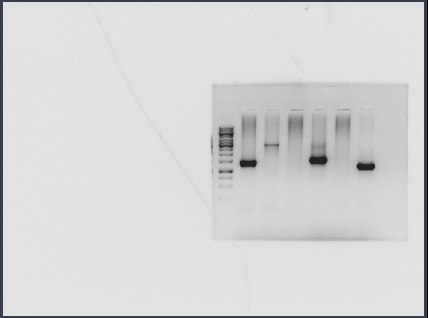

Supplement: Figure 1—source data 2. [file elife-104855-fig1-data2.zip › Figure 1-Source data 2/Figure 1_Panel B.tif]

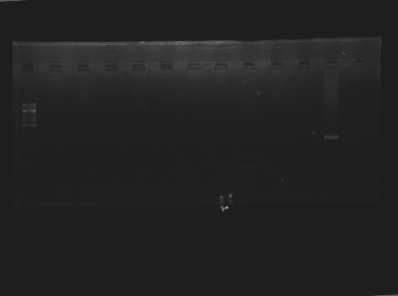

Supplement: Figure 1—source data 2. [file elife-104855-fig1-data2.zip › Figure 1-Source data 2/Figure 1_Panel C adgf-.tif]

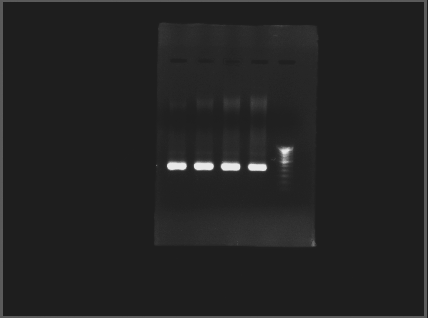

Supplement: Figure 1—source data 2. [file elife-104855-fig1-data2.zip › Figure 1-Source data 2/Figure 1_Panel C rnlA.tif]

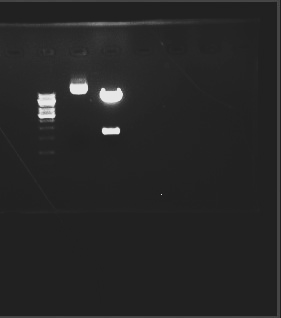

Supplement: Figure 4—source data 2. [file elife-104855-fig4-data2.zip › Figure 4-Source data 2/Figure 4_Panel B.tif]
