# Supplementary material for: Extracellular adenosine deamination primes tip organizer development in Dictyostelium
Source: eLife. 2025 Dec 17;14:RP104855. doi: 10.7554/eLife.104855 (PMC12711200; doi:10.7554/eLife.104855)
Supplement: Figure 4—source data 1. [file elife-104855-fig4-data1.zip › Figure_4_Source_data_1.pdf]

Figue 4-Source data 2_Gel image with labelled bands

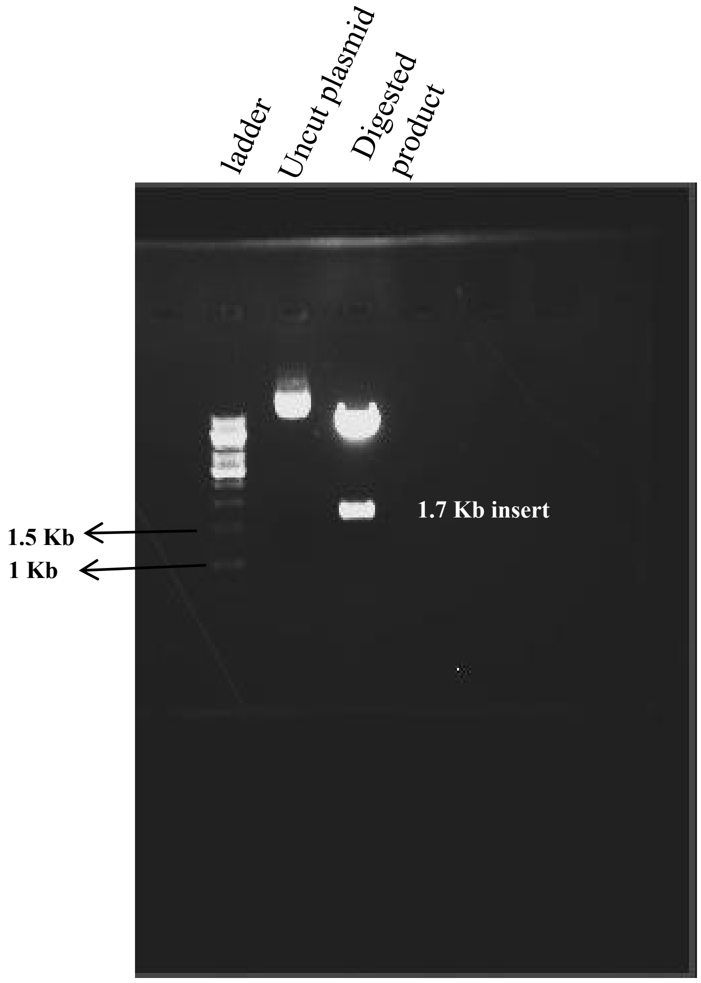

Original gel corresponding to Figure 4, panel B. Thermo Fisher ladder was used.
