# Supplementary material for: Extracellular adenosine deamination primes tip organizer development in Dictyostelium
Source: eLife. 2025 Dec 17;14:RP104855. doi: 10.7554/eLife.104855 (PMC12711200; doi:10.7554/eLife.104855)
Supplement: Supplementary file 1. — Table A: Various deaminases annotated in D. discoideum. Table B: Primers used for adgf semi-quantitative PCR. Primers used for adgf overexpression and vector construction. Table D: Primers used for real-time PCR. [file elife-104855-supp1.pdf]

Table A

Various deaminases annotated in *D. discoideum*

Gene	Gene ID
2-aminomuconate deaminase	DDB_G0275081
adenosine deaminase	DDB_G0287371
adenosine deaminase acting on tRNA 1	DDB_G0278943
adenosine deaminase, tRNA-specific	DDB_G0288099
adenosine deaminase-related growth factor	DDB_G0275179
AMP deaminase	DDB_G0292266
CMP/dCMP deaminase, zinc-binding domain-containing protein	DDB_G0282255 | DDB_G0271914 | DDB _G0286161 | DDB_G0288019
cytidine deaminase	DDB_G0292096
cytidine deaminase-like protein	DDB_G0278841
cytidine/deoxycytidylate deaminase zinc-binding domain-containing protein	DDB_G0292096
dCTP deaminase	DDB_G0293580, DDB_G0268194
glucosamine-6-phosphate deaminase	DDB_G0278873,<br>DDB_G0286195
guanine deaminase	DDB G0277743
porphobilinogen deaminase	DDB_G0284697
putative arginine deaminase	DDB_G0289195
serine deaminase	DDB_G0272787
threonine deaminase	DDB_G0277245
glutamine synthetase	DDB_G0276835,DDB_G0295755
glutamate dehydrogenase	DDB_G0280319, DDB_G0287469
Glutaminase	DDB_G0291984
Allantoicase	DDB_G0280267
Aconitase	DDB_G0279159
Arginine deiminase	DDB_G0272182
Formimidoyl transferase-cyclo deaminase	DDB_G0287977
Fatty acid amide hydrolase	DDB_G0275967

Table B

Primers used for *adgf* semi-quantitative PCR

Primer	Sequence
P1	CCGAAGCTTAAAATGTTTTTAAAGTTTA
P2	ATAGAAATGAATGGCAAGTTAG
P3	GTTGAGAAATGTTAAATTGATCC
P4	TGGATGAGCACGCATATCAG

Table C

Primers used for *adgf* over expression and vector construction

Primer	Sequence
adgf OE FP	CCGAAGCTTAAAATGTTTTTAAAGTTTA
adgf OE RP	GGCGGTACCTTAAATATTTGAATAAGTATTA

Table D

Primers used for real time-PCR

Primer	Sequence
adgf FP	GTGGTGTATGATGCAATGGTAATG
adgf RP	TGGATGAGCACGCATATCAG
ada FP	GAAACGGGTTAACTAAAGAGCAAG
ada RP	TGGATCATCAGAGGTTGAAGAAG
adat FP	ACCAATTTCAGGAGAGTGGAC
adat RP	TTCCTAAACACCTATTACCAGTACC
ada-tRNA FP	GAACAAGACACGCAGAACTTG
ada-tRNA RP	GCACATCAAACATGGCTCTAC
countin FP	CAACCGGTAATGCTTTTGGT
countin RP	CACAAACGAGAGCTGACA
smlA FP	TGGATTACACCATGTTCAGCA
smlA RP	CCGACTGAAACTGATGCTTTGG
acaA FP	CATTCTAGAGGCGGTATTGGC
acaA RP	GGAGAAAATGTCTGATTTCGCTT
carA FP	ATGTTGGGTTGTATGGCAGTG
carA RP	AGGGAAACCACCATTGACAG
pdsA FP	CCATTGGGTACAACTGGTGGA
pdsA RP	AACTGCCCATGATGGATAGGT
regA FP	TAAAGCAACGTTGGCACAAG
regA RP	ATGGTGATTCCATTGCTTCC
pde4 FP	GATCTTGATACACCAATCGAA
pde4 RP	CTTCTGCATCATCTGTACATG
5'nt FP	CAGCTGAACAAGTAGCAATGG
5'nt RP	TGGTGGAAGACTTGATGCTG
cadA FP	TTCCAAGAATTGGCTCAAGG
cadA RP	CATCAACTGCCCATTGAAAA
csaA FP	GCCAAATACAATCGCTGGTG
csaA RP	TGGTTGGTGTGAGATCAAAAGC
ecmA FP	CCAATTAGCTGTCCAAAACC
ecmA RP	GCAATCACCTTTACCTCCTG
ecmB FP	TGATTCATGTTGTTCAACTGG
ecmB RP	TAAATCATCGCCACATTTTCC
pspA FP	CATTGGCCAATCAAAATCCAG
pspA RP	ACAACAGTTGAAGCAGAACC
rnlA FP_qRT	TTACATTTATTAGACCCGAAACCAAGCG
rnlA RP_qRT	TTCCCTTTAGACCTATGGACCTTAGCG
